# Supplementary material for: Are carbon emissions trading and green financial instruments synergistic? -Comprehensive quantitative research based on content analysis
Source: PLoS One. 2024 Mar 7;19(3):e0298601. doi: 10.1371/journal.pone.0298601 (PMC10919601; doi:10.1371/journal.pone.0298601)
Supplement: S1 Data — (ZIP) [file pone.0298601.s001.zip › Data/地级市Z_F工作报告.zip!/╦─┤¿/░═╓╨╩╨/└┤╫╘╣1⁄2╓┌║┼í╛┬φ┐╦╩2╛▌═°í┐_2018.docx]

各位代表：

现在，我代表市人民政府，向大会报告工作，请予审议，并请市政协委员和其他列席人员提出意见。

一、2017年工作回顾

去年以来，面对经济下行的压力和艰巨繁重的发展任务，在市委坚强领导下，在市人大、市政协监督支持下，我们紧紧围绕“建设川陕革命老区振兴发展示范区，走出秦巴山区脱贫攻坚绿色发展新路子”的决策部署，团结带领全市人民苦干实干、攻坚克难，经济社会发展取得新成效，较好完成了市四届人大一次会议确定的年度目标任务，本届政府工作实现良好开局。

（一）经济实现稳中有进。坚持稳中求进工作总基调，着力推进供给侧结构性改革，接续打出“1+5+N”政策组合拳，实施企业培育“四百计划”，扎实推进“项目年”工作。全年完成地区生产总值601.44亿元、增长8.1%，地方一般公共预算收入45.53亿元、同口径增长8.8%，城乡居民可支配收入28286元和10946元、分别增长9.0%和9.8%，新增贷款98.9亿元、增长18.2%。绿色产业加快发展，三次产业结构比为15.5:48.8:35.7，其中文旅产业对经济增长贡献率15.8%，工业产值中四大新型工业占比54.4%，规上工业增加值增长10.7%，新种植核桃、茶叶、巴药31万亩。企业培育初见成效，新增入库规上工业企业38家、限上商贸企业107家、规上服务业企业112家、建筑业企业45家、房地产开发企业11家、省级以上农业产业化龙头企业6家，巴山牧业、圣泉水务先后在“新三板”挂牌。项目工作再创佳绩，完成全社会固定资产投资1370.63亿元、增长14.7%，290个市级重点项目完成年度投资890亿元，190个、总投资4600多亿元的重大项目纳入四川省川陕革命老区振兴发展重点项目库。城乡消费增势明显，完成社会消费品零售总额324.23亿元、增长12.8%，住宿餐饮业增长12.1%，游客接待量和旅游收入分别增长21.1%、26.1%，电子商务交易额增长21%。

（二）脱贫攻坚再战再胜。锁定“两不愁、三保障、四个好”目标，相继发起春季攻势、夏季战役、百日攻坚、冬季攻势，新完成13个片区连片开发，全市9.58万人脱贫、215个贫困村退出，贫困发生率下降到4.5%，巴州区、恩阳区摘帽完成省级考核验收。建成市级“四好村”245个，易地扶贫搬迁和土地政策助推脱贫攻坚的巴中模式、四好村“五建五评五比”机制等成果在全国推广。群众收入普遍增加，新发展2.19万“五园”经济户，9.4万个贫困家庭劳动力实现稳定就业，政策兜底12.23万人。安全住房竣工入住，新开工易地扶贫搬迁住房38534套、建成32779套，改造农村危房21483户。义务教育保障到位，新改造农村义务教育薄弱学校142所，帮助2124名残疾适龄儿童入学，救助家庭困难中小学生18.87万人，“三免一补”政策全面落实。基本医疗实现全覆盖，49.4万贫困人口全部纳入医疗扶助范围，新扶助贫困人口13.62万人次，县域内住院看病费用自付比例控制在10%以内，因病致贫人口减少36.9%。

（三）城乡建设势头强劲。围绕构建现代生态新型城镇体系，大力推进以绿兴城、以文兴城、以游兴城、以产兴城和以彩靓城，城乡发展条件持续改善，城镇化率达到40.1%、提高1个百分点，巴城中心城区建成区面积56平方公里、常住人口65万人，成功创建国家卫生城市、国家森林城市。基础设施不断改善，恩阳机场航站楼全面开建、机场快速通道路基形成，巴广渝高速公路全线通车，巴陕高速公路南江至关坝段建成通车，巴万高速公路加快建设，汉巴南铁路巴中至南充段完成可研等前期工作；红鱼洞、黄石盘水库完成年度建设任务，天星桥等4座中型水库枢纽工程完工；杨家坝110千伏输变电站等4个电网项目建成投运，城西220千伏、周家坝110千伏输变电工程开工，实施农网改造项目356个；新建成4G基站833个、宽带乡村677个。城市建设扩容提质，“一控规两导则”编制规程正式出台，城市开发边界、生态红线和水面蓝线基本划定，完成中心城区增绿添彩设计方案和市县绿地系统规划，巴城新增彩化面积6万平方米、绿地面积2万平方米；麻柳湾至巴河大桥滨河路改造基本完成，南环线贯通指日可待，12.5公里地下管廊启动建设；市中心医院南坝院区（一期）、成都中医大附属医院巴中分院、市体育馆游泳馆、市残疾人康复中心（托养中心）建成，市社会福利院试运行，市法检公业务用房、市政务服务中心、市体育中心、市惠民帮扶中心、市气象灾害监测预警中心、地矿救灾“一基地三中心”等项目主体工程完工，市精神病康复院开工。镇村建设成果丰硕，巴中获得全国唯一“新农村建设综合标准化示范市”称号，驷马镇入选全国特色小镇，化成镇入选全省特色小镇，光雾山镇入选全国最美森林小镇，空山镇入选省级森林小镇；新建中心村21个、聚居点357个、幸福美丽新村261个，新获批67个省级传统村落，成功创建1个中国最美休闲乡村。产业基础切实加强，城市工业园区不断优化，落户企业明显增多；特色农业规模化发展，新建高标准农田22.72万亩，完成土地整理26.16万亩，新增特色农业基地21.53万亩；现代服务业产业基地大幅增加，国家全域旅游示范区创建正式启动，光雾山—诺水河世界地质公园创建通过联合国教科文组织专家实地评估，光雾山AAAAA景区创建通过国家旅游资源评审，川陕革命根据地红军烈士陵园AAAAA景区创建纳入省红色旅游“饮水思源”行动，玉湖入列国家水利风景区，新建成7个省级森林康养基地。恩阳区连续两年获评全省县域经济发展先进县。

（四）改革开放有序有力。坚持政府与市场良性互动、改革与开放协同发力，巧干实干，借力发展，重点改革事项和战略合作事项取得新的进展。“放管服”改革创新突破，依法取消或下放行政权力391项，“双随机一公开”执法检查实现全覆盖，组建市企业投资促进协调服务中心，设立行政审批、金融信贷、公共服务三个“一站式”服务窗口，马上办、网上办、一次办事项分别达到51%、77%、93%，全程电子化登记和多证合一、一照一码全面实行，新登记市场主体2.56万户。国资国企和投融资改革深入实施，完善现代企业制度，初步形成以管资本为主的制度体系；组建市文旅集团和市交投集团，设立川陕革命老区振兴发展基金；落地建设PPP项目23个，其中7个列入省示范项目，入选个数、投资额均居全省第一；市级监管国有企业实现营业收入15.2亿元；资本市场直接融资59.24亿元；巴中市成为全省唯一同时实施农业产业发展融资风险保障金试点和“政融保”项目的市（州）。农村产权制度改革持续深化，颁发农村土地承包经营权证75万余本，完成52个村房地一体登记试点、60个乡镇集体产权制度改革，基本完成村级集体资产清理核实工作；全市新增流转耕地15.23万亩、林地6万亩，供销合作社托管土地2万亩；跨市流转增减挂钩节余指标4405亩、耕地占补平衡指标4.42万亩，交易金额21.9亿元。开放合作取得新的成效，成都—巴中协同发展战略全面实施，与广元市、中国茶叶研究所达成新的战略合作协议，与国网四川电力签订巴中电网建设合作协议；新落地招商项目271个，新建成招商项目204个，到位资金534.5亿元、增长9.4%。创新创业扎实推进，成立巴中市农林科学研究院，新增国家高新技术企业4家、省政府授牌企业技术中心3家、省级工程技术研究中心3个，新增省级科技企业孵化器2家，申请专利突破1000件大关，建成市级院士（专家）工作站8家；回引4100余人返乡创业、创办投资规模100万元以上经济实体808个，平昌县入列国家第二批“双创”示范基地，通江县获批国家第三批支持农民工等人员返乡创业试点县。

（五）民生福祉不断改善。回应群众关切，办理民生实事，公共财政支出的72.8%投入到民生领域，省定民生目标全面完成，市自选10件民生实事深入推进。群众生产生活条件持续改善，全市新建村组道路1254公里，新解决16万农村居民饮水安全，改造城镇危旧房棚户区1.635万套，完成5个城镇老旧小区供配电设施改造，同类别用电实现同价；巴城投放共享单车6500辆，公交优先、出租汽车改革和网约车发展同步推进；城市低保月标准、农村低保年标准和城乡特困人员供养月标准分别提高40元、120元、100元，在全国异地住院就医初步实现即时结算。城乡新增就业3.46万人，城镇登记失业率4.3%。社会事业加快发展，新（改、扩）建城区义务教育学校7所、农村教师周转房1347套，化解城市超大班734个，义务教育实现免试就近划片招生和县域内“四统一、全覆盖”，恩阳、平昌县域义务教育基本均衡发展通过国家评估认定；医联体、乡村卫生一体化改革扎实推进，公立医院全面实行药品采购“两票制”，开展新生儿健康筛查2.9万余人次、妇女“两癌”筛查5.5万余人次；成立巴文化研究院，成功举办第五届巴人文化艺术节，优秀曲艺剧目《望红台•守望》获文化部嘉奖，建成广播“村村响”工程303个、电视“户户通”3.66万户，完成巴中电视台高清改造；实施贫困村农民体育健身工程80个。社会治理成效显著，《巴中市红军文物保护条例》《巴中市城市道路交通秩序管理条例》颁布实施，“法律七进”扎实开展，“平安巴中”建设深入推进，立体化治安防控体系不断完善，依法化解重点突出信访积案200余件，安全生产、食品药品安全、治安管理、应急处突、金融风险防范等工作责任落实、措施有效，生产安全事故数、死亡人数分别下降35%和38%，圆满完成一系列重大时期维稳任务，社会形势总体平稳。国防动员、民兵预备役和双拥工作扎实有效，统计、保密、档案、地方志、民族、宗教、外事、台侨、关心下一代、老龄、气象、人防等事业都取得了可喜成绩。

各位代表！在去年的市人代会上，市政府主要领导承诺督办4件民生实事，目前办理情况是：新建城市公厕14座，如数完成目标任务；新建城区停车泊位10555个，超目标555个；新（改）建城区公办幼儿园7所，超目标2所；原计划新建的41个乡镇污水处理站已建成12个、在建17个，余下的因去年4月上级政策调整，目前正在以市为单位统一打捆申报入库，之后将抓紧实施。

（六）生态文明切实加强。省委绿化全川决策部署有效落实，环境保护“党政同责、一岗双责”压紧压实，各级政府研究部署环保工作成为常态，首部实体性地方法规《巴中市城市饮用水水源保护条例》正式实施，中央环保督察交办和省环保督察反馈问题年度整改任务全面完成。污染防治“三大战役”首战告捷，扬尘污染、露天焚烧、油烟污染和“散乱污”等空气污染行为得到治理，成为全省唯一新增的“环境空气质量达标市”；“河长制”全面落实，在全省率先实行“河道警长制”，主要河流出境断面水质达到或优于Ш类标准，城市集中式饮用水源水质达标率100%、乡镇超过90%；农业面源污染治理全面加强，循环农业试点建设初见成效。生态保护持续加强，自然保护区监管措施不断强化，完成永久基本农田划定329.4万亩，新增退耕还林7.8万亩、营造林52万亩，森林覆盖率达到58.5%；完成地质灾害避险搬迁2568户，连续六年实现“零伤亡”。全市立案查处环境违法案件163件，未发生较大以上环境污染和生态破坏事件。

各位代表！在推动整体工作的同时，针对发展中面临的各种困难和问题，市政府自我加压、勇于担当，打出“八个一批”工作组合拳，取得明显成效。在推进一批滞后项目上，10个未开工的中央预算内项目全面开工，13个进展缓慢的市级重点项目新完成投资11.91亿元。在理顺一批管理体制上，组建市旅游发展委员会和秦巴产品质量检验检测中心，明确南龛文化产业园管理新模式，供销、安监、城管执法、林业综合行政执法、土地储备及国有林场等通过改革实现了体制与形势相适应、职责与任务相匹配、权力与责任相对等。在放大一批基金效益上，“十大基金”整合为“七大基金”后，总规模达到11.89亿元，累计撬动信贷资金投放28.26亿元。在引导一批民间投资上，全市完成民间投资646.61亿元、同比增长19.3%，对投资增长贡献率达到61.6%。在集聚一批金融产业上，全市新引进金融机构6家，实现全国股份制银行、扶贫银行分（支）行零的突破，秦巴金融街完成场地平整、正在积极推进首批3家金融机构总部入驻相关工作。在疏解一批老城城市功能上，18个老城功能疏解项目全面启动，巴城汽配街、大佛寺加油站搬迁工作全面完成，巴城江北水果市场、西城蔬菜市场批发业态搬迁工作加快推进，南池市场达成整体搬迁协议。在化解一批国土住建领域信访积案上，涉及延期交房、“两证”办理、合同纠纷等5类20件信访积案有序化解，其中建筑面积300余万平方米、涉及2万余户的23个停工（半停工）项目恢复施工。在打击一批违规违法行为上，开展非法营运和互联网金融风险专项整治，大力查处“假私非超”，破获非法吸收公众存款和集资诈骗案件17件、挽回经济损失1.9亿元，拆除各类违法建设19万平方米，帮助1.28万名农民工依法追回欠薪超过1.3亿元。

在总结成绩的同时，我们必须清醒认识到，全市经济社会发展仍然面临一些困难和问题：经济总量小、人均水平低、结构不优、效益不高的状况没有根本改变，经济增长主要依靠投资拉动，消费提振基础不稳固；基础设施和公共服务欠账较大，脱贫攻坚任务繁重，城乡发展和居民收入不平衡不充分；一些重点领域矛盾和问题比较突出，办事依法、遇事找法、解决问题用法、化解矛盾靠法的法治良序尚未根本形成；“四风”问题在一些地区和行业不同程度存在，官僚主义、形式主义现象时有发生，人民群众对此不满意。这些问题，必须在今后工作中切实加以解决。此外还需要说明的是，创建全国文明城市虽然连续几年付出艰辛努力、取得了阶段性成果，但由于硬件设施、运营秩序、市民文明素养等方面原因，未能获得成功。尽管如此，巴中人民崇文尚善的精神不会止步，文明城市创建的脚步不会停歇！

一年来的工作成绩，是市委坚强领导、市人大和市政协监督支持以及全市广大干部群众凝心聚力、克难攻坚、奋勇拼搏的结果。在此，我代表市人民政府，向全市人民群众，向人大代表、政协委员和各民主党派、工商联、无党派人士、各人民团体，向所有关心支持巴中发展改革的同志们、朋友们表示衷心的感谢！向驻巴部队、武警官兵和民兵预备役人员致以崇高的敬意！

二、2018年工作安排

2018年是落实党的十九大精神的开局之年，是决胜全面建成小康社会、实施“十三五”规划承上启下的关键一年，也是全市上下乘势而上、凝心聚力建设川陕革命老区振兴发展示范区的全面起步之年，做好全年工作意义重大。

政府工作总体要求是：以习近平新时代中国特色社会主义思想为指导，认真落实党的十九大、省委十一届二次全会、市委四届五次全会和中央、省委、市委经济工作会议精神，坚持党的领导，坚持稳中求进工作总基调，坚持新发展理念，按照高质量发展的根本要求和市委“六个走在前列”、实施“六大突破”、强化“六个推进”部署要求，以供给侧结构性改革为主线，以脱贫攻坚为统揽，以绿色崛起为路径，统筹稳增长、调结构、促改革、惠民生、防风险，打好“三大攻坚战”，为建设川陕革命老区振兴发展示范区、走出秦巴山区脱贫攻坚绿色发展新路子而努力奋斗。

综合考虑发展取向和目标导向，2018年全市经济社会发展的预期目标是：地区生产总值增长7.8%，全社会固定资产投资同口径增长10%，社会消费品零售总额增长13.5%，地方一般公共预算收入增长8%以上，城乡居民人均可支配收入分别增长9%、11%，城镇登记失业率控制在4.4%以内。实现上述目标，要重点抓好以下工作：

（一）扭住供给侧结构性改革，大力发展绿色产业

巴中当前已处在产业加快转型升级的关键时期。要按照“经济质效提升走在前列”的要求，坚持质量第一、效益优先，以供给侧结构性改革为主线，以打造旅游经济强市为龙头，构建三产联动的绿色经济体系，打造以绿色经济为特色的新兴增长极。

大力发展全域旅游。围绕加快建成全国知名旅游目的地和森林康养目的地，依托绿色生态资源和红色文化优势，大力发展生态旅游、红色旅游、乡村旅游、森林康养。突破推进国家全域旅游示范区创建，年内完成《巴中市全域旅游发展总体规划》编制，争取光雾山AAAAA景区、光雾山—诺水河世界地质公园命名，建成5个省级以上森林康养基地，川陕革命根据地红军烈士陵园创建AAAAA景区争取达到省级验收评估条件，全省红色旅游融合发展示范区建设取得实质进展。加快完善旅游配套设施，开工建设米仓大道（诺水河至光雾山一级公路），系统规划布局机场至景区、高速公路出口至景区、城镇至景区的快速通道和AAAA景区之间连接线，启动巴城骑游道建设，新建游客接待中心4处、自驾游营地（露营地）3个，实施好“厕所革命”。深入推进重点景区建设，推动旅游产品转型升级，开发一批休闲度假旅游产品，推出一批“体验游”精品线路，打造一批旅游风情小镇，培育一批特色旅游村落，打响“巴山民宿”品牌。深度发掘特色文化元素，合力开发特色旅游商品，创作影视歌戏，办好节展赛会，建设购物站点，突出精准营销，推动音乐演艺、文化创意、影视传媒与旅游产业融合发展，综合提升巴中旅游影响力和市场竞争力，力争全年接待游客2900万人次、实现旅游收入246亿元，文旅产业对经济增长贡献率达到17%。

突破发展特色农业。坚持质量兴农、绿色兴农，围绕建成秦巴山区绿色农产品生产基地和生物医药基地，以深化农业供给侧结构性改革为抓手，优品种、建基地、精加工、促营销、创品牌，推动特色农业转型升级。实施茶叶、核桃、巴药、生态养殖“四大农业产业行动计划”，划定粮食生产功能区和重要农产品生产保护区256万亩，新建高标准农田21万亩，新（改、扩）建现代农业标准化基地5万亩，种植茶叶、核桃、巴药23万亩，发展生态养殖场（园）60个。实施“区域品牌+企业品牌+产品品牌”战略，推动农产品精深加工，推进有机产品认证示范市创建，提升“巴食巴适”“巴中云顶”区域公用品牌影响力，增强通江银耳、南江黄羊、青峪猪、平昌白酒、三强药业、好彩头等“巴中产”“巴中造”产品竞争力，积极培育省级名牌产品，新增国家驰名商标1个。实施“订单农业+家庭生产”电商全覆盖计划，加快构建优质农产品县、乡、村一体化流通服务体系，让巴中优质农产品走进大市场。坚持农旅融合发展，推进园区景区社区同建，高标准打造田园综合体，新建产业融合园区5个、休闲农庄10个、农业主题公园3个，创建省级现代农业产业融合示范园区2个。

积极发展新型工业。坚持围绕绿色抓工业，大力发展食品饮料、生物医药、新能源新材料、电子信息产业，力争三强中药配方颗粒、特兴智能LED芯片等30个项目竣工投产，石英晶片及手机陶瓷配件生产（一期）等20个项目开工，规上工业增加值增长10.5%。坚持片区化统筹、规模化布局，积极发展“飞地工业”，推动经开区创建国家级园区、其他园区创建各类省级园区（基地）。实施重点工业企业培育培优计划，加快推进企业规范化公司制改制，新增规上工业企业20户，建成工旅融合示范企业5家。

加快发展现代服务业。推进市场体系建设，打造4条特色餐饮街区，巴中西部国际商贸城居然之家、南江亿联家居电商城建成投运，启动经开区公铁综合物流园建设，做深做实巴中恩阳机场临港产业园项目前期工作。加快实施国家（省）级电子商务进农村示范项目，推动通江县、平昌县示范项目通过国家验收，建成乡镇电商服务站20个、村级电商服务点200个。持续培育消费热点，做大做强“巴中枣林鱼”“南江黄羊大酒店”等特色餐饮品牌，支持企业开办一批直营店、加盟店、网上店，推动家政服务、文化娱乐、体育运动等新兴消费加快发展。积极发展加工贸易，力争实现加工贸易出口额3亿元以上。推动金融产业健康发展，加快建设秦巴金融街，巴中农商银行总部迁建开工；积极争取筹建地方法人银行和市级统一法人农村商业银行，年内引进金融机构4家以上；用好用活川陕革命老区振兴发展基金，增强金融服务产业、孵化企业功能，力争1家企业上市、5家企业在新三板或新四板挂牌。实施服务业企业培育培优计划，新增限上商贸流通企业和规上服务业企业100户，培育电商骨干企业10家、第三方物流重点企业10家。

（二）下足“绣花”功夫，打好脱贫攻坚战

当前，巴中面临精准脱贫示范与同步全面小康的双重任务。要按照“脱贫攻坚决胜走在前列”的要求，持续下足“绣花”功夫，突破推进精准扶贫精准脱贫，确保年内73851人脱贫、168个贫困村退出、南江县摘帽，加快建设全国精准脱贫示范区。

推进脱贫攻坚对标达标。聚焦“两不愁”，因户制宜发展“五园”经济，同步发展长效增收产业；扎实开展劳动技能、实用技术、素质提升培训，实现转移就业2.5万人次；坚持“兜底线、保基本”，保持“两线合一”，实现贫困群众应保尽保、应扶尽扶。聚焦住房安全有保障，易地扶贫搬迁54128人，改造贫困户危房2万户。聚焦义务教育有保障，精准落实“三免一补”等15项教育资助政策，确保无贫困家庭孩子因贫辍学。聚焦基本医疗有保障，精准落实“十免四补助”等健康扶贫政策，建立健全重病重残儿童救助机制，进一步降低因病因残致贫发生率。抓好脱贫成果巩固提升。抓好已脱贫对象后续扶持，坚持脱贫不脱政策、脱贫不脱帮扶，吸纳已脱贫户融入“四大农业特色产业”，增强造血功能，提升就业创业能力，确保稳定脱贫不返贫。深入开展“回头看、回头帮”，精准实施动态调整，及时将新增贫困对象纳入帮扶范围，不漏一户一人。巩固提升已建成92个片区连片开发成果，新完成8个片区连片开发，实现100个片区道路互通、产业连片、服务共享，整体提升贫困村和非贫困村脱贫水平。

强化脱贫攻坚工作保障。完善资金多元化投入模式，用好用活“四项扶贫基金”，整合财政涉农资金25亿元，撬动金融投入，吸引社会投入，确保全年扶贫投入不低于120亿元。统筹贫困村与非贫困村，按照“保基本、补短板”的基本思路，优化完善脱贫攻坚项目库和扶贫专项方案，将扶贫资金落到项目上、用到关键处，产生实实在在的效益。压实贫困村“五个一”、非贫困村“三个一”帮扶责任，沉下去、贴着帮，深化励志扶贫、企业扶贫、网络扶贫、公益扶贫等多元化扶贫模式，形成全社会扶贫大格局。深化脱贫攻坚领域作风建设，强化暗访督查、专项检查、问题整改和责任追究，确保脱贫攻坚群众认可、经得起历史检验。

（三）扩大有效投资，推进基础设施建设

稳增长首要的是稳投资。要按照“基础设施跨越走在前列”的要求，突出主攻方向，着眼互联互通，持续扩大优质增量供给，加快推进交通、水利、能源、通讯设施建设，同步扩大产业、民生和社会领域项目投资。

扎实抓好项目工作。巩固全市上下大抓项目的良好态势，持续抓好向上争取工作，专班跟促已纳入川陕革命老区振兴发展项目库的重大项目落地实施。深耕国家政策投向和市场取向，精心包装一批基础设施、产业、民生和科技项目，力争全年储备项目700个左右、总投资2000亿元左右。实施市级重点项目280个，强化要素保障和质量、安全管控，确保年内完成投资734亿元、竣工项目70个。用好用活优惠扶持政策，推动民间投资持续稳定增长。

打好第三轮交通大会战。突出“联网、加密、升级”主攻方向，突破推进以“6631”为骨干的交通项目建设，年内完成投资125亿元，实现恩阳机场通航、巴陕高速公路全线通车、巴城南环线投运，巴万高速公路巴中至通江段形成通车能力，开工建设苍溪至巴中高速公路巴中段、巴广渝高速经开区互通和巴城北环线东段，力争汉巴南铁路巴中至南充段开工、镇广高速公路通江广纳至广安段启动招商。完成普通国省干线公路升级改造150公里，建成村道联网路1122公里、村内通组路3340公里，建成渡改桥9座，建制村通客车率达到90%。加快S204线通江县城—诺水河一级公路、通江和南江通用机场、巴中城市轨道交通等项目前期工作。

推进重点水利工程建设。围绕“2+5”在建水利工程、“2+6+7”前期水利项目，梯次推进重点水利工程建设。年内完成二郎庙、双桥、天星桥3座水库蓄水验收，红鱼洞、黄石盘、湾潭河水库分别完成投资总量的70%、40%、50%，加快江家口、青峪口水库等项目前期工作；实施150座小型病险水库除险加固；巩固提升39万农村居民饮水安全保障能力。

构建稳定共享的能源信息网络。推进电能替代工程，完成替代电量1300万千瓦•时，建成一批电动汽车充电设施。加快实施重点电力项目，年内实现巴中城西220千伏、通江周家坝110千伏输变电工程竣工投产，开工建设巴中城西220千伏变电站配套工程、巴达铁路巴中牵引站110千伏改接供电工程等8项输变电工程。推进以数字网络、智慧巴中为支撑的现代通讯设施建设，打造全市共建共享共用的电子政务云、基础数据库和公共信息服务平台，建成智慧城市运营管理中心，新建4G基站600座，升级“宽带乡村”496个。

（四）坚持以人为本，统筹提升城乡建设水平

巴中当前的城镇化率远低于全国全省平均水平。要坚持以人为核心，以提升城镇化水平和建设美丽乡村为重点，以实施乡村振兴战略为主线，突破推进四级城镇体系建设，努力把城市做优、集镇做特、农村做美，推动城乡同发展共繁荣。

优化提升城市功能。大力推进市中心城区建设，实施城市“双修”工程，优化提升巴城老城区6平方公里控规及城市设计，完成中心城区农贸市场布点、2条历史文化街区保护、3个交通堵点疏解、全市新能源汽车停车位充电桩布局规划编制和高压走廊规划修编；坚持新区园区景区三区同建，统筹巴城三区协同发展，加快推进兴文新区、恩阳新城基础设施和公共服务配套建设，持续推进老城功能疏解完善，推动巴州城区高中有序外迁、市中心医院部分功能向南坝院区转移，完成江北水果市场、西城蔬菜市场批发业态和南池市场搬迁以及莲花街风貌打造；推进增绿添彩，启动实施巴河回风大桥至大佛寺段景观打造，加快九寨山彩色植物园、义阳山山地公园建设，加快推进津桥湖、陇桥、大溪口等湿地公园建设，新增巴城绿化面积9万平方米、彩化面积3万平方米；加强市政基础设施建设，启动江北柳津桥至廊桥滨河健身步道工程，打通老观桥路、江北滨河路巴河大桥至中坝段、江南滨河路桑园坝至南杨大桥段等3条次干道，疏通一批交通堵点。推动三县加快城市副中心建设，突破发展县域经济，形成全市“一盘棋”统筹发展格局。扎实推进国家环保模范城市、国家级生态文明建设示范市、国家园林城市及全国文明城市创建工作。稳步发展建筑房地产业，加大绿色建筑推广力度，积极发展装配式建筑和旅游地产、康养地产；突出住房居住属性，强化分类调控，优化房产供给结构，规范建设开发秩序，从严打击违法建设，推动房地产市场健康发展。

推动镇村一体建设。巩固国家新农村建设综合标准化示范市建设成果，高度重视乡镇对农村发展的龙头和引擎作用，统筹推进镇村规划，完成5个特色镇城镇设计、180个村和300个聚居点规划、67个新增国家和省级传统村落保护规划编制。推行“特色镇+幸福美丽新村”建设模式，重点打造特色镇5个、幸福美丽新村260个，新争取4-5个镇纳入全省百镇建设行动试点，争创全省第二批特色镇2个，新建成中心村21个、聚居点150个，提升打造恩阳古镇，在有条件的地方建设一批村史馆、农耕文化博物馆。持续推进农村人居环境治理，改造农村危旧土坯房5.85万户。

实施乡村振兴战略。按照“产业兴旺、生态宜居、乡风文明、治理有效、生活富裕”总要求，统筹谋划乡村“五位一体”建设，编制出台乡村振兴战略实施规划，配套完善城乡融合发展的体制机制和政策体系，清除阻碍要素下乡的各种障碍。推进农村土地“三权分置”改革，推进农村不动产统一登记，完善农村土地管控机制。完成60个乡镇集体产权制度改革，完善县（区）乡村三级农村产权交易市场体系，新增流转耕地10万亩以上、林地5万亩以上；搭建集体产权抵押融资平台，开展农业产业发展融资风险保障金试点，实现产权抵押融资贷款10亿元以上。构建现代农业产业体系、生产体系、经营体系，大力培育新型农业经营主体，新增省级以上农业产业化龙头企业3家，新发展农民专业合作社100个、家庭农场50个，通过订单生产、就近务工、入股分红把农民聚在产业链上，促进小农户和现代农业有机衔接。探索完善自治、法治、德治“三治一体”的乡村治理体系，年内建成省级“四好村”110个、市级“四好村”260个；在50个村开展社区建设试点，建成三星级社区11个。建立农业职业经理人制度，加大农业产业带头人扶持力度，大力培育新型职业农民。

（五）从人民群众关心的事情做起，全力保障和改善民生

保障和改善民生必须抓住人民最关心最直接最现实的利益问题。要按照“民生福祉改善走在前列”和“社会治理创新走在前列”的要求，既量力而行，又尽力而为，一件事情接着一件事情办，一年接着一年干，加快弥补民生短板，坚决打好防范化解重大风险攻坚战，切实维护社会公平正义，持续增强人民群众获得感幸福感安全感。

抓好民生工程和民生实事。持续加大民生投入，推动省下达民生目标任务逐项落实落地。全力办好市自选10件民生实事，新完成197个村农网升级改造、20个城镇老旧居民小区供电设施改造，新建城区公共停车泊位10000个、乡镇污水处理站43座、乡镇加油站11座、蔬菜生产基地6500亩，新增设一批巴城老旧住宅电梯，新（改）建城乡公办幼儿园10所、城区公厕30座、农贸市场53个。

大力发展社会事业。优先发展教育事业，持续改善办学条件，着力解决中小学生课外负担重、择校热、大班额、超大校区等问题，建成乡镇标准化中心校30所，化解义务教育阶段大班额587个、基本消除66人以上超大班，巴州区、通江县县域义务教育基本均衡发展通过国家评估认定；推进高中阶段普及攻坚计划，实施新高考“选课走班”教学改革；积极推进本科院校争取筹建工作；扩大职业教育规模，提高职业教育质量，深化产教融合和校企合作，结合地方产业办专业，增强职业教育服务地方经济社会发展的能力；强化师德师风建设，加强校长队伍管理，大力实施乡村教师支持计划，培育一批名师名校。着力深化健康巴中建设，年内市第一人民医院试运营，市精神病康复院完成主体工程，积极组建巴中城市医疗集团，推进平昌县医疗共同体改革试点和全市乡村卫生一体化改革，规范基层医疗机构管理；培育一批名医名科名院；全面推行医保支付方式改革，加快推进异地就医全国联网即时结算；积极构建多层次的养老服务体系、统筹城乡的社会救助体系，建成村（社区）残疾人康复站120个，建成妇女之家、儿童之家200个，新建社区家长学校或家庭教育服务指导站100个，实施好“童伴计划”、妇女两癌筛查救助等公益项目。广泛开展全民健身，承办好环中赛等重大赛事，年内建成市体育中心。大力支持工会、共青团、妇联、科协等开展工作，加快发展老龄、残疾人等事业，扎实抓好档案、地方志、统计、民族、宗教、外事、侨务、对台、保密、地震、人防、综治、保险等工作，做好第四次全国经济普查工作，推动区县争创新一轮省级双拥模范城，积极支持国防和军队建设，抓好征兵、退伍军人管理、优抚优待等工作。

推动文化事业繁荣兴盛。健全公共文化服务体系，推进秦巴群众文化活动中心和巴中博物馆项目，启动南龛文化产业园红博馆、飞霞阁、兵器展场布展工作。加强文化遗产遗迹保护，推动出台《巴中市石窟保护条例》，编制红军石刻标语群保护规划，实施“川陕苏区首府”再现工程，接续申办巴人文化艺术节，打造一批反映巴人巴风巴韵及巴中时代精神的文艺精品，加快建设晏阳初故里小镇，举办晏阳初文化研讨活动，打响“巴文化中心、川陕苏区首府、晏阳初故里”三张文化名片。深化“书香巴中•全民阅读”活动，全面实施文化场馆免费开放，建成城市书房6个、贫困村文化室215个。大力实施文化惠民工程，引进精品剧目10场次以上，组织送文化下乡220场次。加快“智慧广电•高清巴中”项目建设，实施好180个广播“村村响”、2万户电视“户户通”工程。

打好防范化解重大风险攻坚战。着力防范金融风险，组建地方金融管理机构，建立“审批、监管、处置”一体的地方监管体系，推进社会信用体系建设，健全信息披露和诚信档案制度、失信联合惩戒机制和黑名单制度，化解不良贷款，打击非法集资，严防发生系统性金融风险。防范化解政府债务风险，做好存量政府债务置换，严格限定债务率较高的县（区）举债规模、举债程序和资金用途，切实加强监督管理。健全矛盾纠纷多元化解体系，依法规范信访秩序。强化安全生产和食品药品监管，建设“智慧安监”“智慧消防”“智慧交通”“智慧食药”等平台，建成5个安全体验中心、6个省级安全社区，加强消防公共设施建设，推动餐厨废弃物无害化处理在全市餐饮行业实现全覆盖，确保全年无重特大生产安全和食品药品安全事故发生，成功创建省遏制重特大安全生产事故示范市。强化社会治安综合治理，推进综治中心、雪亮工程和公共安全视频监控体系建设，持续整治非法营运，严厉打击“两抢一盗”“假私非超”、网络通信诈骗等各类违法犯罪，依法保护农民工合法权益，保持社会大局和谐稳定。大力推进依法治市，深入开展“七五”普法，加强公共法律服务实体、网络、热线等“三大平台”建设，初步形成覆盖城乡、功能完备、便捷高效的公共法律服务体系，提升依法治理水平。

（六）深化改革创新，加快构建开放型经济格局

改革开放是经济社会发展的不竭动力。要按照“改革开放推进走在前列”的要求，坚持把改革创新作为先导性措施，推动市场无形之手和政府有形之手协同发力，促进创新主体和市场主体倍增，努力打造开放高地、投资洼地、创业福地。

深化重点领域和关键环节改革。全力做好国省改革事项承接工作，全面启动川陕革命老区综合改革试验区建设，推动各项改革任务落实落地。纵深推进“放管服”改革，运用“互联网+政务服务”，实现全省一体化政务服务平台在市县乡全覆盖，持续提升“马上办、网上办、一次办”政务服务水平。深化财税体制改革，加快完善现代财政制度，健全政府预算、绩效评价、财政监督、风险防控体系，深入研究“小财政支持大发展”的机制和办法，调整优化财政支出结构，做大做强川陕革命老区振兴发展基金。扎实抓好国资国企改革，加强国有资本监管，防止国有资产流失；推动投融资公司转型发展，使之成为重大基础设施、重大产业和重大社会事业项目的投资主体、融资载体、营运主体和承债主体；支持国有资本和国有企业做强做优做大，市文旅集团和市交投集团实质运营，培育骨干企业6家。深入推进统计管理体制改革，推动依法治统，强化统计数据质量管理。持续深化供销社综合改革，改造提升中心基层社10个，恢复基层供销社25个。

促进开放合作项目落地。拓展开放的范围和层次，主动融入国家“一带一路”和长江经济带，深度承接成渝、珠三角等经济圈产业转移和市场辐射。推进成都—巴中协同发展，抓好设立成都产业转移集中区、开通“空巴通”和景区直通车、流转耕地占补平衡和增减挂钩节余指标、建立成都农村产权交易所巴中分所、建立在蓉医疗机构巴中远程会诊平台、委托定向培训专业技术人才等合作事项落实落地。推进广元—巴中战略合作，加快推动G542、S301、S302、S303等普通国省干线公路改造升级。不断深化企地合作，包装策划超亿元产业项目150个、超5亿元项目30个，引进重大产业项目25个以上，到位各类招商资金不少于550亿元；办好中国秦巴山区（四川•巴中）绿色农林产业投资贸易洽谈暨第27届中国西部商品交易会，省级以上重大平台活动签约项目100个以上。推进与知名高校、科研院所的合作，争取一批具体项目落地。

推进大众创业万众创新。加快建设省级生物医药高新技术产业化示范基地，创建国家农业科技园区达到验收标准，增强市农林科学研究院创新转化能力，培育5家国家高新技术企业，引进推广50项科技成果，新申请专利1100件。加快推进“双创”示范基地建设，备案国家“星创天地”和省级“众创空间”2－3家。扎实推动“巴山优才”计划，引进培育一批创新创业科研团队、急需紧缺人才和高层次人才，加快推进“人才公寓”建设。持续深化“返乡创业五年行动计划”和“高校毕业生就业创业促进计划”，全年回引返乡创业人员3000人以上、创办投资规模100万元以上的经济实体不少于600家，引领200名以上大学生创新创业。实施推进民营经济发展行动计划，扶持一批重点民营企业，形成保护企业、尊重企业家的浓厚氛围。

（七）守护绿水青山蓝天，全面加强生态文明建设

绿色生态是巴中最具竞争力的名片。要秉持“绿水青山就是金山银山”的理念，坚持生态优先、绿色发展，加快形成节约资源和保护环境的空间格局、产业格局、生产方式和生活方式，努力建设美丽巴中。

打好污染防治攻坚战役。持续打好“蓝天保卫战”，巩固空气质量达标成果，强化大气污染联防联控，确保城市空气质量优良天数率90%以上。坚决打好“碧水保卫战”，全面落实河长制，深入实施“五大行动”，强化流域环境综合整治，加强城乡集中式饮用水源保护，确保城市及乡镇集中式饮用水水源地水质达标率分别为100%和90%以上、市域地表水水质保持Ⅲ类及以上标准。全力打好“净土保卫战”，完成农用地土壤污染状况详查。

加大生态系统保护力度。强化中央和省环保督察发现反馈问题整改，集中开展“九项治理行动”，加快生态环境监测网络建设，持续提升市域环境质量。编制环境准入负面清单，严格执行生态保护红线、永久基本农田、城镇开发边界三条控制线，加强全市1个国家级、5个省级自然保护区管理。坚持生态环境保护与绿色经济发展同步推进，持续开展“九大绿化行动”，完成人工造林25万亩、退耕还林3万亩，森林覆盖率达到59%，推动林业碳汇交易实施。

加强环保能力建设。加快推进垃圾污水处理项目，启动7个城乡非正规生活垃圾填埋场整治，开工巴中固废循环经济产业园和城乡垃圾污水处理设施PPP项目。加强防灾减灾救灾能力建设，治理水土流失面积100平方公里，建成新一代天气雷达，启动国家基本气象站搬迁和66个地灾点区域自动气象站建设，完成地灾避险搬迁1000户。

提升环保监管水平。改革生态环境监管体制，全面落实环保机构监测监察执法改革各项部署，健全网格化环境监管体系，开展领导干部自然资源资产离任审计，建立市场化多元化生态补偿机制、市县环保督察机制和国土资源执法监管共同责任机制。保持环境执法高压态势，依法打击环境违法行为，严防重大环境污染和生态破坏事件发生。推进节约型机关、绿色家庭、绿色学校、绿色社区创建和“绿色出行”行动，增强大众环保意识和自我监管能力。

各位代表！以上七个方面的工作，事关建设川陕革命老区振兴发展示范区伟大实践的顺利起步，事关同步全面小康宏伟目标的如期实现。一分部署，九分落实。让我们携起手来，一步一个脚印朝前走、一项一项抓落实，共同奏响振兴发展的时代强音！

三、政府自身建设

去年，我们坚持“以人民为中心”的发展思想和“五个第一”工作导向，以“行政效能提升年”活动为载体，为市民解决了一批难事、办成了一批实事，提升了政府的执行力和公信力。今年，我们将以“不忘初心、牢记使命”主题教育为契机，加强“五大建设”，提升“五种能力”，持续打造人民满意政府。

（一）加强思想政治建设，提升维护核心能力。深入学习党的十九大精神，自觉以习近平新时代中国特色社会主义思想武装头脑、指导实践、推动工作。树牢“四个意识”，把维护习近平总书记核心地位作为最大政治，坚决维护党中央权威和集中统一领导。自觉向习近平总书记看齐，向党中央看齐，向党的理论和路线方针政策看齐，向党中央决策部署看齐，确保中央大政方针和省委、市委决策部署在政府系统落地落实。

（二）加强法治政府建设，提升依法行政能力。深入实施《法治政府建设实施纲要》，持续培育法治精神，不断强化“法无授权不可为，法定职责必须为”的法治观念，凡事以法为先、坚持于法有据。牢固掌握政策体系，按政策推动发展，按政策促进改革，按政策服务群众，不断提升执行政策的水平。修订完善政府规章，自觉接受人大法律和工作监督、政协民主监督，认真听取民主党派和工商联、无党派人士意见，推动法治政府建设再上台阶。

（三）加强创新政府建设，提升克难攻坚能力。始终保持专注发展定力，树立为巴中振兴发展攻坚克难、不胜不休的雄心壮志，主动担责、人人担当。不断强化改革创新意识，结合巴中实际大胆改革、先行先试，蹄疾步稳、锐意创新，积极探索巴中加快发展新路子。咬定青山不放松，推动各项改革举措在各条战线各个领域落地生根、开花结果，面对未来有所建树、展现作为。

（四）加强民生政府建设，提升服务群众能力。着力强化为民担当，正确面对民生投入不足、居民收入不高、全市整体贫困的不争事实，始终把人民对美好生活的向往作为奋斗目标，把持续增强群众获得感幸福感作为最大责任。深度回应群众关切，用好用活“三信两直播一热线”，扭住群众需求持续办好民生实事。充分尊重群众主体地位，依法维护群众知情权、参与权、表达权、监督权，集智聚力共建美好巴中。

（五）加强廉洁政府建设，提升拒腐防变能力。坚持挺纪在前，严守政治纪律和政治规矩，按本色做人，按角色做事。严格落实“两个责任”，依法实施审计监督，支持监察巡视巡察监督，惩治不干净的人，查处不廉洁的事。锲而不舍抓作风，驰而不息纠“四风”，大兴实干文化，培育担当精神，旗帜鲜明为“老实人”加油鼓劲，坚决处置为官不为、阳奉阴违，努力维护风清气正、崇廉尚实、干事创业、遵纪守法的政治生态。

各位代表！经广泛征求意见，今年市政府主要领导亲自督办的5件民生实事是：新建城区公共停车泊位10000个，新（改）建城乡公办幼儿园10所、城区公厕30座、城市书房6个、农贸市场53个。

各位代表！我们正处在一个伟大的时代，蓝图令人鼓舞、使命催人奋进。让我们紧密团结在以习近平同志为核心的党中央周围，更加坚定地把思想和行动统一到党的十九大精神和省委市委决策部署上来，奋力谱写新时代巴中发展的美好篇章。我们坚信，站在1.23万平方公里绿色大地上，秉承4万多英雄儿女的革命遗志，传承建地设市24年来的奋斗精神，聚合全市380万干部群众的磅礴力量，建设川陕革命老区振兴发展示范区的蓝图一定会变为现实，巴中的明天一定会更加美好！
